# Fluoxetine results in misleading conclusions on fish behavior

**DOI:** 10.1002/ece3.7797

**Published:** 2021-06-28

**Authors:** Malgorzata Grzesiuk, Alicja Pawelec

**Affiliations:** ^1^ Department of Biochemistry and Microbiology Institute of Biology Warsaw University of Life Sciences (SGGW) Warsaw Poland; ^2^ Department of Hydrobiology at Biological and Chemical Research Centre Faculty of Biology University of Warsaw Warsaw Poland

**Keywords:** antidepressant, environmental concentration, *Gobio gobio*, *Neogobius fluviatilis*, personality, recovery

## Abstract

Fluoxetine is an antidepressant medicine causing relaxation and mood improvement in people, with silencing certain personality traits in some cases. The question arise if such phenomena can be observed in nontarget organisms such as fish. Fluoxetine affects fishes behavior; however, it is not known if the medicine affects its “personality.” This study aimed to evaluate the reaction of the invasive *Neogobius fluviatilis* and native *Gobio gobio* individuals to fluoxetine at environmental concentration of 360 ng/L. We prepared three variants of the experiments: (a) behavioral trials with unexposed fishes, (b) behavioral trials with the same fishes after 21 days of fluoxetine exposure, and (c) behavioral trials with the same fishes after 21‐day depuration period, that is, without fluoxetine. The fishes reaction time (RT), that is, difference in time spent on reaching food with and without the necessity of overcoming the obstacle, was analyzed. Additionally, the personality, bold or shy, traits of each fish individual, was assigned. The results indicated that environmental concentrations of the antidepressant influenced RT. The average RT of the fishes cultured with fluoxetine was by 7‐min shorter in comparison with the nonexposed control. Share of individuals exposed to fluoxetine assigned as bold raised to 71.4% in comparison with 46.4% in nonexposed control. This sheds new light on wild fishes behavior caught from freshwater. Environmental concentrations of the antidepressant influenced the time of fishes reaction and share individuals assigned as bold. Moreover, 21‐day recovery lasting might be not enough to get fluoxetine effect on fishes.

## Graphical abstract

Environmental concentrations of the antidepressant influenced time of fish reaction and share individuals assigned as bold. 21‐day recovery lasting might not be enough to get rid of fluoxetine effect on fish.

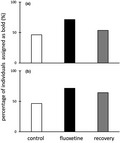

## 1 INTRODUCTION

Freshwater ecosystems contain 41% of the world's known fish species (Daily, 1997). Unfortunately, freshwater biodiversity faces many threats. Aquatic environments, and especially small bodies of the water, are currently one of the most degraded types of ecosystems (WWF Living Planet Report, 2020). Pharmaceuticals are a threat to modern biodiversity. The reason is permanent presence of pharmaceutical substances in the freshwater ecosystems (Hughes et al., 2013). Among these medicines, there is the commonly used and increasingly prescribed antidepressant, fluoxetine (Prozac), a selective serotonin reuptake inhibitor (Schultz et al., 2010; Silva et al., 2012). The medicine is prescribed in the case of depression, anorexia, bulimia, obsessive and compulsive disorder, panic disorder etc., and became the third most frequently consumed group of pharmaceuticals (Mojtabai & Olfson, 2011). Two main sources of antidepressants in wastewater are sewages from households and psychiatric hospitals. Fluoxetine excreted by humans is only partially removed by current wastewater treatment processes (Du et al., 2014; Kookana et al., 2014) and over months exhibits minimal degradation or transformation in sewage (Redshaw, 2008).

It was already shown that environmentally relevant concentrations of pharmaceuticals affect so‐called nontargeted organisms such as algae (e.g., Grzesiuk et al., 2018) and invertebrates (e.g., Grzesiuk et al., 2019; Grzesiuk, Mielecki, et al., 2018; Grzesiuk et al., 2020). Environmental concentrations of medicine are often low (e.g., for fluoxetine, maximal concentration found was 596 ngL^‐1^; Hughes et al., 2013), and pharmaceuticals typically are designed to show the biological effect at low doses acting on a physiological level that usually is evolutionary conserved across taxa (Arnold et al., 2014). As much as 65%–86% of human drug targets are conserved in 12 diverse fish species (Brown et al., 2014).

Fishes are the most important organisms shaping aquatic ecosystems and trophic webs by being both prey and predator, prey to some species and predator to the others. As prey, fishes are known to change their behavior in various ways as a response to the presence of predators (review, Lima & Dill, 1990; Magurran & Seghers, 1990). Some species decrease their activity when responding to the predator (Mathis & Smith, 1993), others switch feeding to hiding and schooling (Magurran & Seghers, 1990), or avoid dangerous habitat (Keefe, 1992; Pettersson & Brönmark, 1993). Some planktivorous fishes also switch to feeding at night instead of during the day to avoid predation.

The effect of fluoxetine on different kinds of behaviors of fishes, such as mating, feeding, predator avoidance, and aggression, has been already tested (e.g., Barry, 2013; Bertram et al., 2018; Di Poi et al., 2013; Fursdon et al., 2019; Lynn et al., 2007; Martin, Bertram, et al., 2019; Martin, Saaristo, et al., 2019; McCallum et al., 2017; Pelli & Connaughton, 2015; Perreault et al., 2003; Peters et al., 2017; Saaristo et al., 2017; Weinberger & Klaper, 2014). Our first goal was to confirm these results. We hypothesized that 21‐day exposure to environmental concentrations of fluoxetine significantly affects fishes behavior.

Observed the animal behavior, we can conclude that not all individuals within a species react in the same way when exposed to the same type of conditions, even if they are of the same age, sex, and place of origin. A relatively new branch of experimental behavioral ecology is the study of innate and acquired intraindividual variation in animal behavior which have now be performed on a variety of species (Reale et al., 2007; Sih et al., 2004). The phenomenon has been described using several different terms, such as “personality,” “temperament,” and “behavioral syndromes.” “Animal personality” is a term that covers several components of the behavior of an individual organism, such as boldness, sociability, and aggressiveness (so‐called “personality traits”). The personality traits are specific tendencies in behavior, for example, constant tendency to explore new areas and boldness. These traits differ among individuals, are constant over time, and dictate how each individual responds in different environmental contexts (Reale et al., 2007). According to the behavior under consideration, individuals are arranged along a behavioral gradient, such as the bold/shy continuum (Wilson et al., 1993). The process of forming a personality trait remains poorly understood in an individual, but most probably, it has a complex physiological and hormonal foundation (Koolhaas et al., 1999). Moreover, described in the 1970s individual differences in behavior have been ignored in behavioral research considered as insignificant “noise” and a source of random statistical error (Dall et al., 2004). It was only at the beginning of the 21st century that researchers, for example, Gosling (2001), began to look closer at differences in behavioral categories within species, and the phenomenon is now recognized as a natural factor of adaptive importance.

Fluoxetine is the most active ingredient in antidepressant Prozac (Brooks, 2014). Persons taking it show relaxation and improvement of mood. In some cases, it may silence certain personality traits of the patients, making them calmer. Therefore, we started to consider whether such a phenomenon can be observed in a nontargeted organism like fishes.

It is well established that fluoxetine affects behavior of fishes, but the influence on the “personality” that is strictly associated with behavior has not been recognized. In one type of personality, wide spectrum of behavior can be observed. The question arises if the pharmaceutical effect is strong enough to significantly change behavior in such a way that assignment to personality is false. Our second hypothesis states that long time exposure to fluoxetine under environmental concentrations masks innate personality trait of fishes. To test our research hypothesis, we conducted an experiment where fishes were exposed to environmentally relevant fluoxetine concentrations and the behavior and personality traits were analyzed.

## 2 MATERIALS AND METHODS

### 2.1 Animal description and experiment conditions

The behavior of two species of fish, invasive Monkey Goby *Neogobius fluviatilis* and native Gudgeon *Gobio gobio*, was observed. Both species occur in the same European habitats: in lakes and large‐to‐medium‐sized rivers and streams, on sandy or muddy bottoms with shallow water up to 2 m deep. They also show similar biology. Reaction to the danger of individuals of both species and the effect of antidepressant on the reaction to the danger were monitored. Additionally, we analyzed the recovery effect, that is, how animals previously exposed to pharmaceutical respond to dangerous situations.

Thirty *N*. *fluviatilis* individuals were caught in Narew River, Poland. Also, 30 *G*. *gobio* originated from fishes ponds were bought from breeders. We selected 14 fish from each species. *Neogobius fluviatilis* had similar body length (4–6.5 cm total body length) and weight (2–4 g). *Gobio gobio* had similar body length (4–6 cm total body length) and weight (2.5–4 g). Monkey Gobies and Gudgeons were housed separately in 5‐L tanks, one individual per tank. Each tank had sandy bottom (100 g of sterilized sand per tank) preferred by both species. All tanks were aerated. Half of the water (2.5 L) was changed every 2 days. Fishes were fed once every 2 days with their natural food, namely *Chironomidae* larvae.

Aware of the fact that it is impossible to completely avoid habituation, to minimize this risk as much as possible, we kept wild fishes in the laboratory for a very short time before starting the experiment. On the other hand, we also needed to avoid the risk of testing sick fishes, which can interfere with the results. We waited the shortest possible time, that was 2 weeks, allowing to observe and exclude any sick fishes and to minimize the risk of habituation.

**FIGURE 1:**
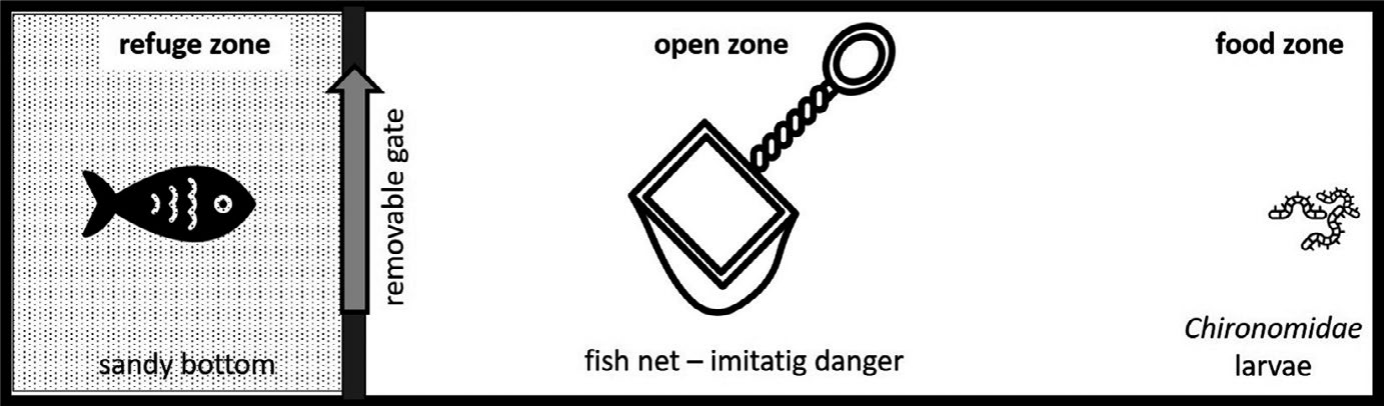
The experimental system (icone source: The Noun Project (https://thenounproject.com/))

After 14 days of acclimatization under above‐described conditions, we measured reaction time and personality traits (boldness/shyness) of each individual using special experimental system. The system included a rectangular aquarium (L 100 cm × W 30 cm × H 20 cm). The aquarium was divided into three zones: (a) refuge with the sand bottom where the fishes were able to hide, (b) an open space without the possibility of hiding and possible danger, and (c) a zone with the food alive *Chironomidae* larvae dispensed. The refuge zone was separated from the rest of the aquarium by a removable gate (Figure 1).

Experiments were conducted for 3 days in a row. Before the experiment, the water temperature was noted. Each day at the same time the same fishes individual was tested. At the beginning of each experiment, fishes were placed in the “refuge” zone and the same portion of food was introduced into the third zone. The behavior of the fishes in the system was recorded using two cameras located on tripods connected to the computer disk where the videos were archived, and to the monitor, so that the experiment could be controlled without unnecessary experimenter interference.

First, 5 hr before starting the experiment, each fish was placed in the system and left for 30 min to learn it, and then, they went back to their pre‐experimental tanks. Bold or shy type of personality means the way animals respond to danger but not the new situation (Reale et al., 2007). Next, on the same day, fish was again placed in the experimental system, and after the gate of the safety zone was opened, the time the fish individuals reached food was measured. This was “0” experimental day, without threatening the fish. On the first and second experimental days, the fish were also threatened three times by immersing the fishnet in front of them in an open space zone. To get to the third zone and get the food, fish had to take a risk, swim out of the safe zone, and overcome the obstacle. The same procedure we used on the second experimental day. The time the fish individuals reached food was measured. Reaction time was measured in two replicates (day 1 and day 2). Here, we show the results from day 1 only. In analysis, both replicates were used since the statistical analysis did not reveal time effect on reaction time RT (repeated‐measure ANOVA: Wilkins Lambda = 0.999; *F*_(2,155)_ = 0.005; *p* = 0.994).

Series of experiments described above were conducted three times: (a) fishes cultured without antidepressant (control treatment), (b) the same individuals exposed to fluoxetine for 21 days (fluoxetine treatment), and (c) again, the same fishes cultured without fluoxetine for another 21 days (recovery treatment) were tested

Analyzing the innate behavior of individual, especially personality traits, has been always difficult. One of the main problems is the lack of typical “control treatment” as researchers are using in other types of experiments. While testing innate behavior of a specific animal individual, it is impossible to have control treatment classically understood: You would have to get some kind of clone of this individual, not only genetically, but also a 100% copy of whole innate behaviors of this animal, which is impossible. The only way to compare data is to compare previous individual behavior when the animal is not subjected to the examined factor (here: the presence of fluoxetine) with current animal behavior, when the individual is subjected to the examined factor, as we did in our experiments.

### 2.2 Pharmaceuticals

Fishes were exposed to commonly used antidepressant pharmaceutical fluoxetine, known as Prozac. This antidepressant is prescribed more often each year (e.g., by 15% in the USA; OECD Health Statistics 2014). Fluoxetine (SIGMA) was a minimum 98% pure. The concentrations of fluoxetine in the culture medium were chosen following literature reports on the concentrations detected in several freshwater ecosystems (Huggett et al., 2003; Hughes et al., 2013), that is, 360 ng/L (max. concentration found 596 ng/L). Fluoxetine solution was added to the medium to receive wanted concentration. The experimental media were prepared with a fresh dose of the pharmaceutical to avoid antidepressant decay.

### 2.3 Analyzed parameters

The reaction time of fish individuals from both species and different treatments (control, fluoxetine, recovery) was analyzed. We considered the reaction time as the difference in time of reaching food of individuals threatened and not threatened. Personality traits: Boldness/shyness of each individual has been assigned after reaction time analyses. Animal's personality is define as continuum, and it is unchanged during life. Animal assigned as bold/shy in each behavioral observation can show a little different result, but similar enough to be assign to a certain personality type. Based on Kortet et al. (2014) work, we proposed to use median reaction time of species to split and assigned fishes to two personality traits: bold and shy. Median of reaction time was calculated for control animals together from both species. Fishes showing reaction time longer then its median were considered as shy whereas fishes showing reaction time equal and shorter then median were considered as bold. The median obtained from control treatment was used to assign personality for individuals from fluoxetine and recovery treatment.

### 2.4 Statistical analysis

Statistical analyses were performed using Statistica 13.3 software. Distribution fitting analysis showed normal distribution of the data. The reaction time and personality traits of fish individuals were tested using repeated‐measure ANOVA followed by Tukey HSD post hoc analysis (*p* < 0.05). Differences in personality were tested in three groups: (a) control with fluoxetine treatment, (b) fluoxetine with recovery treatment, and (c) recovery with control treatment.

## 3 RESULTS

### 3.1 Reaction time

**FIGURE 2:**
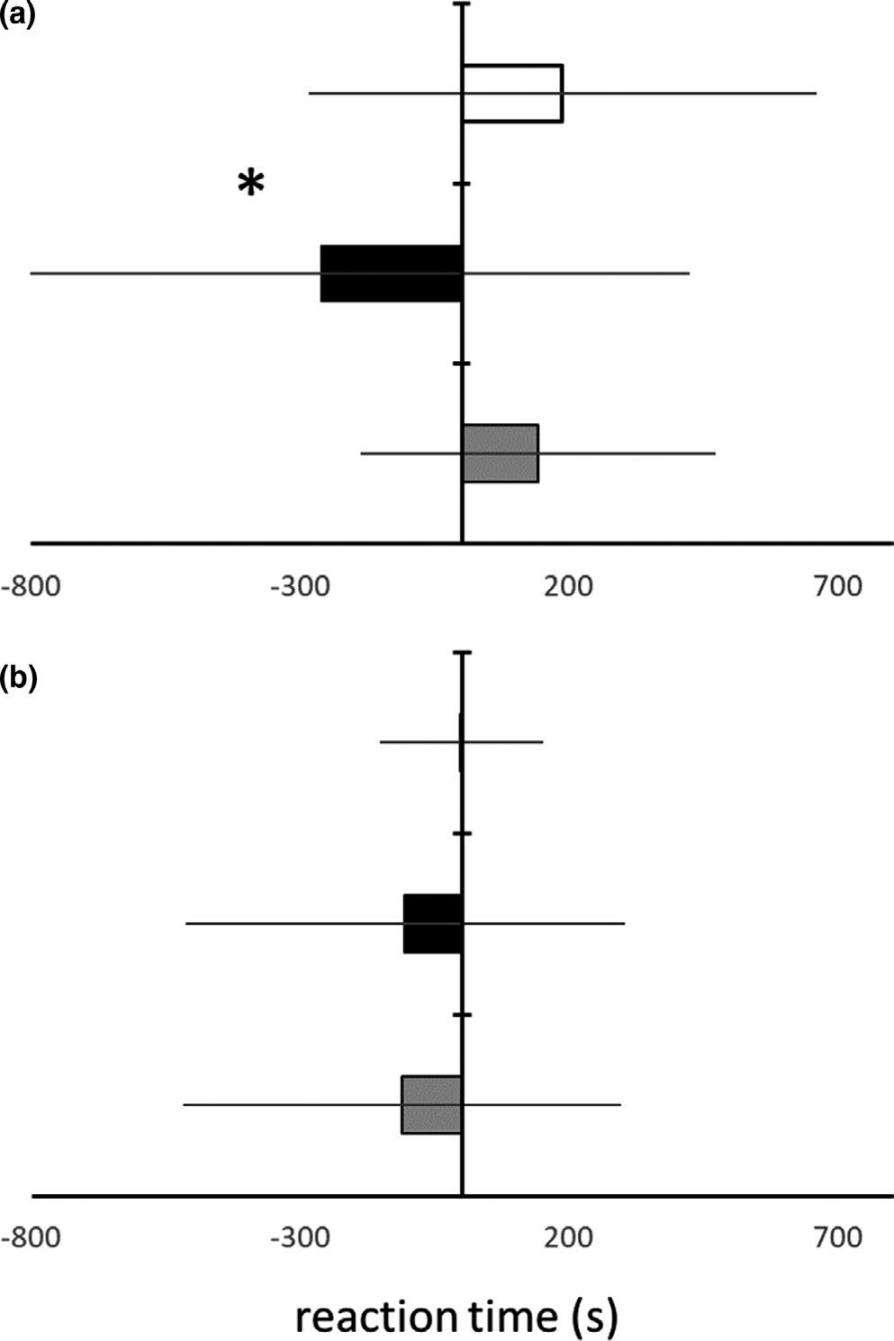
The reaction time (average ± *SD*) of reaching food by *Neogobius fluviatili*s (a) and *Gobio gobio* (b). (Difference in RT between threatened and not threatened individuals.) Fishes cultured without fluoxetine (white bar), the same individuals exposed to fluoxetine for 21 days (black bar), again the same fishes cultured without fluoxetine for another 21 days (gray bar). Star indicates significant differences among treatments (repeated‐measure ANOVA, post hoc Tukey HSD)

Analysis of the reaction time (RT) revealed a statistically significant effect of species (repeated‐measure ANOVA: Wilkins Lambda = 0.248; *F*_(4,310)_ = 234.7; *p* < 0.0001). Moreover, analysis of the RT showed a statistically significant pharmaceutical effect (repeated‐measure ANOVA: Wilkins Lambda = 0.930; *F*_(2,155)_ = 2.9; *p* = 0.023) (Figure 2). Post hoc analysis (Tukey HSD) revealed significant differences in RT between *N*. *fluviatilis* animals cultured without antidepressant and the one exposed to the pharmaceutical for 21 days. An average RT for *N*. *fluviatilis* cultured without pharmaceutical was 187 s. An average RT of the same fish cultured with fluoxetine was 7.5 min shorter. Moreover, the *N*. *fluviatilis* from recovery treatment also show statistically significant differences in RT in comparison with fishes exposed to pharmaceutical (Tukey HSD; Figure 2a). The fish from recovery treatment showed more than 6.5‐min longer RT in comparison with the fish cultured with fluoxetine. The “recovery” fish did not differed in RT from *N*. *fluviatilis* control. Both groups differ in reaction time by 50 s. In comparison, RT of native *G*. *gobio* was not effected by pharmaceutical (Figure 2b). Reaction time of *G*. *gobio* cultured without pharmaceutical was around 0 s. An average RT of the same fish cultured with fluoxetine was almost 2 min shorter. Surprisingly, on average RT of fish from recovery treatment was more than 3.5% shorter than the one for the fish exposed to fluoxetine (Figure 2b).

### 3.2 Personality traits

**FIGURE 3:**
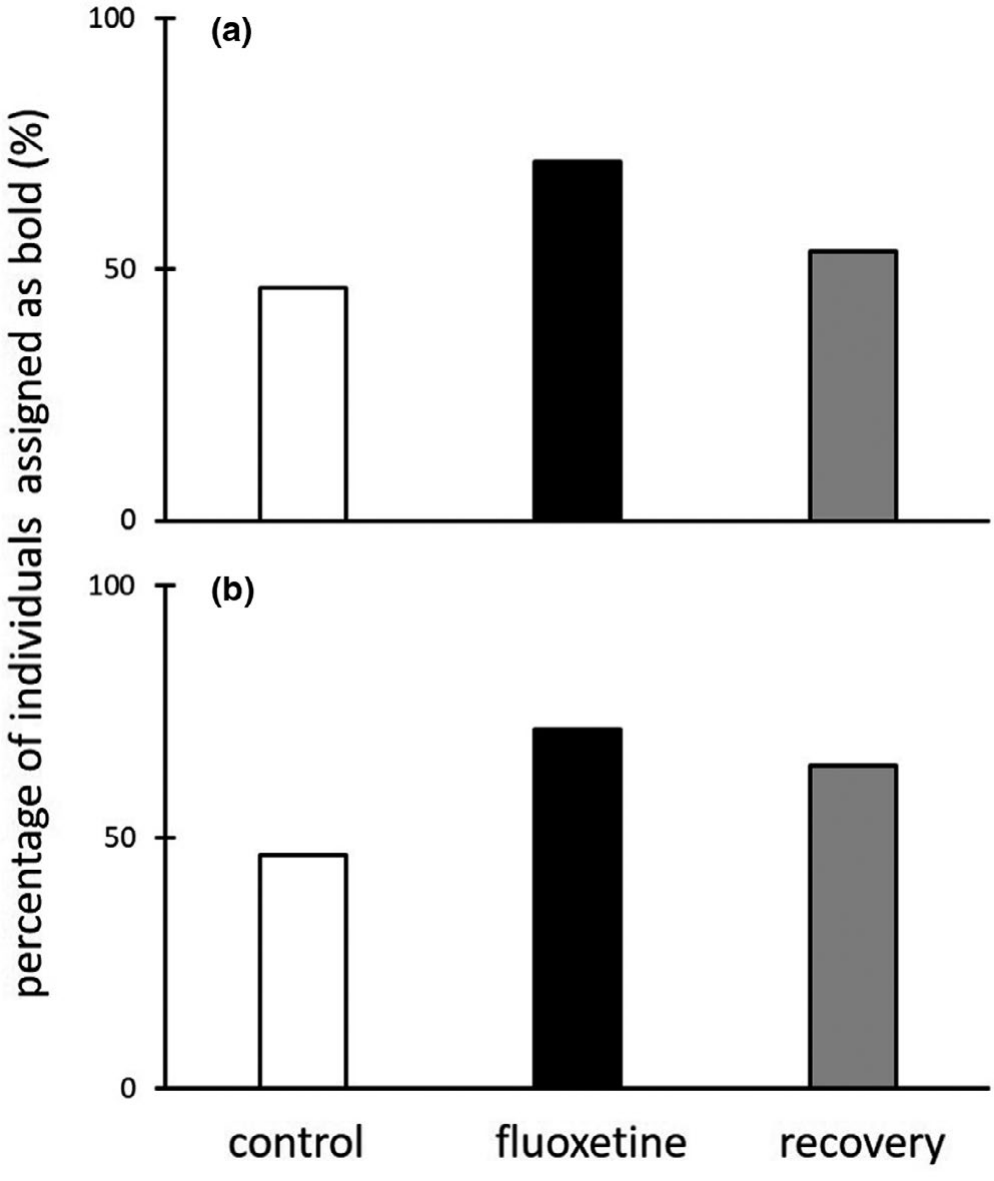
The percentage of *Neogobius fluviatilis* (a) and *Gobio gobio* (b) assigned as bold. Fishes cultured without fluoxetine (white bar), the same individuals exposed to fluoxetine for 21 days (black bar), again the same fishes cultured without fluoxetine for another 21 days (gray bar)

Statistical analysis of personality between control and the fishes cultured with fluoxetine showed significant pharmaceutical (repeated‐measure ANOVA: Wilkins Lambda = 0.941; *F*_(2,103)_ = 3.2; *p* = 0.044) and species effect (repeated‐measure ANOVA: Wilkins Lambda = 0.240; *F*_(2,103)_ = 163.4; *p* < 0.0001). Personality trait analysis showed increase in the share of individuals assigned as bold in both fluoxetine and recovery treatment (Figure 3). Fish from both species exposed to fluoxetine had 71.4% bold individuals in comparison with 46.4% cultured without the pharmaceutical (Figure 3).

When comparing assigned personality of control animals with recovery individuals and recovery with fluoxetine‐exposed animals, statistical analysis did not reveal significant pharmaceutical effect. In recovery treatment, 53.6% and 64.2% of *N*. *fluviatilis* (Figure 3a) and *G*. *gobio* (Figure 3b) individuals, respectively, were assigned as bold.

## 4 DISCUSSION

### 4.1 Reaction time

The results of our study confirmed that 21‐day fishes exposure to fluoxetine at environmental concentrations significantly affects their behavior (Figure 2). Our finding is in the line with the results of latest research. Only recently, papers describing realistic fluoxetine concentrations in the environment and even 15 months long exposure were published (e.g., Martin, Bertram, et al., 2019; Martin, Saaristo, et al., 2019; Vera‐Chang et al., 2019; Wiles et al., 2020).

The second aspect of changes in behavior is recovery time which is the time necessary for fishes to return to its state before the exposure. Gaworecki and Klaine (2008) stated that serotonin levels did not recover after 6 days in *Morone saxatilis*. Dzieweczynski et al. (2016) confirm this information. In their study, one‐week recovery period did not turn *Siamese* male behavior to the form before the exposure. In our study, the recovery period lasted 21 days. The individual RT after recovery period did not differ statistically from never exposed fishes, and also from individuals exposed to pharmaceutical, indicating that recovery is not completed. This aspect needs further investigation. In human, fluoxetine steady‐state concentrations were achieved within 3–4 weeks of daily dosing (Thompson, 2002; Vaswani et al., 2003; Wong et al., 1995). This is why we decided to expose animals for 21 days. The results showed that at least 3 weeks is necessary to reverse the antidepressant effect. One needs to be cautious. When looking at average RT obtained by *G*. *gobio* from recovery treatment and individuals exposed to pharmaceutical, values are almost the same, that is, −107 s and −111 s, respectively. Fluoxetine tends to accumulate in different organs. It gathers in lungs, liver, brain, etc. On the other hand, fluoxetine shows a long half‐life of 1–4 days (Hiemke & Härtter, 2000). All these aspects, and probably more, are responsible for the necessity of very long recovery time.

### 4.2 The fluoxetine impact on ecosystems

Another intriguing conclusion coming from the obtained results is that even environmental concentrations of fluoxetine can change the functioning of entire ecosystems, not only freshwater. Fishes are the most important organisms shaping aquatic ecosystems and trophic webs by serving at the same time as both, prey to the same species and predator to the others (Fricke et al., 2021). Impacting fishes behavior by changing their reaction to stress, fluoxetine affects perception of environmental stimuli both, antipredator and predator at once. Fish individuals show no proper condition assessment of the environment. When being prey, fishes are more vulnerable to predation from birds and mammals such as otter and American mink (Brzeziński, 2008). This way, fluoxetine can affect the terrestrial ecosystem as well. As mentioned above, fluoxetine accumulates in different organs (lungs, liver, brain) and may transfer along with trophic web; that is, fishes under the influence of fluoxetine will be eaten by the bird mink or otter, which, subsequently, may transfer fluoxetine along again by being prey for eagles, otters, foxes, or wolves (Salo et al., 2008). Moreover, the force of antidepressant impact depends on species, body weight, age, etc., and the same fluoxetine concentration can be lethal to fishes embryos but shows no spectacular effect on large adult predators (also terrestrial once). Maybe fluoxetine is another selecting factor. Confirmation of our theory can be found in a recent Polverino et al. (2021) publication. The authors found strong evidence that fluoxetine impairs adaptive potential to environmental change in fishes.

Responses to the presence of low, detected in the environment, concentrations of pharmaceuticals are generally not included in current risk assessment schemes. The importance of these responses in terms of population survival and ecosystem functioning is poorly understood. Pharmaceuticals can affect populations indirectly, thought the food chain, for example, if a key prey species are negatively affected (Kidd et al., 2014). It is necessary to understand these relationships to discover the broader implications of nonstandard observations on ecosystem health.

### 4.3 Personality traits

Our study focused also on fluoxetine effects on personality traits. By definition, personality traits are specific tendencies in behavior (in our study the boldness) that differ between individuals, and dictate how each individual will respond under different environmental conditions, and in context of this study, most importantly are constant over time (Reale et al., 2007). In other words, personality is unchangeable. When assigning a personality to an animal, we analyze individual behavior. The effect of fluoxetine on behavior is strong enough to mislead researcher in personality assigning. Moreover, even three‐week lasting laboratory culturing of fishes in water without pharmaceuticals can be not enough to obtain reliable results. There is no statistically significant pharmaceutical effect on assigned personality in two out of three tested groups, that is, (a) fluoxetine and recovery treatment, and (b) recovery and control treatment. After 21‐day recovery period, still 7% of *N*. *fluviatilis* and almost 18% of *G*. *gobio* additional individuals in comparison with control animals were assigned as bold. It depends on fish species, size, age, etc., and on the specific subject of this research. We should be aware of possible errors in personality assessment.

### 4.4 Invasion species

Biological invasions are one of “the biggest killers” of world biodiversity (Maxwell et al., 2016). Invasive species are one of the more important causes of native species extinctions (Blackburn et al., 2019), especially in aquatic ecosystems (Gangloff et al., 2016; Leunda, 2010). Among vertebrates, fishes are the most vulnerable group to invasions of alien species (Fricke et al., 2021). Grabowska et al. (2009) and Kottelat and Freyhof (2007) have suggested three main factors most important in determining the effectiveness as an invader of a non‐native species. These species should (a) exhibit a high degree of tolerance to a wide range of ecological factors, such as salinity and chemical pollution; (b) show a reproductive strategy characterized by repeated spawning over the season, nest‐building males and the guarding of eggs by the same males until hatching; and (c) the use of opportunistic feeding strategies. Interestingly, in the present study RT was effected by pharmaceutical in invasive *N*. *fluviatilis* individuals. This situation can be explained by the history of invasion of *N*. *fluviatilis* in Europe. Monkey Goby origin from the Ponto–Caspian region. In this region, the use of antidepressants was and still is not that frequent as in Europe (Park et al. 2014). Low antidepressant consumption should result in a low pharmaceutical presence in aquatic environment. It is possible that *N*. *fluviatilis* could have less frequent contact with antidepressants and this makes individuals of this species less resistant and therefore more susceptible to fluoxetine effects. Unfortunately, the knowledge of pharmaceutical occurrence is poor or absent in some parts of the globe, also in Ponto–Caspian region (Hughes et al., 2013). Due to lack of data, we are not entitled to draw conclusions in this matter.

Martin, Bertram, et al. (2019) indicated that neuroactive pharmaceuticals, such as fluoxetine, can affect aquatic life by causing subtle but important shifts in ecologically relevant behaviors. We can add to this statement that these, at first sight subtle behavior shifts, can influence our description and understanding of ecosystem functioning.

## 5 CONCLUSIONS

1. Our study confirmed that environmental concentrations of fluoxetine can change fishes behavior.
2. When assigning a personality to an animal, we should take antidepressant contamination under the account.
3. Invasion fish species can be more sensitive to antidepressant pollution than native fish species.
4. Current risk assessment schemes should take antidepressant contamination under consideration.

## CONFLICT OF INTEREST

The authors declare no conflicts of interest. The manuscript provides original research data not published or considered for publication elsewhere.

## AUTHOR CONTRIBUTIONS

**Malgorzata Grzesiuk:** Conceptualization (equal); Data curation (lead); Formal analysis (lead); Funding acquisition (equal); Methodology (supporting); Project administration (equal); Resources (equal); Supervision (equal); Validation (lead); Visualization (lead); Writing‐original draft (lead); Writing‐review & editing (lead). **Alicja Pawelec:** Conceptualization (equal); Data curation (supporting); Formal analysis (supporting); Funding acquisition (equal); Investigation (lead); Methodology (lead); Project administration (equal); Resources (equal); Supervision (equal); Writing‐original draft (supporting); Writing‐review & editing (supporting).

## Data Availability

Data set is available online: Dryad https://doi.org/10.5061/dryad.xpnvx0kd7.
